# Supplementary material for: Asian Sand Dust Particles Enhance the Development of Aspergillus fumigatus Biofilm on Nasal Epithelial Cells
Source: Int J Mol Sci. 2022 Mar 11;23(6):3030. doi: 10.3390/ijms23063030 (PMC8955751; doi:10.3390/ijms23063030)
Supplement: Supplementary file 1 [file ijms-23-03030-s001.zip › ijms-1607937-supplementary.pdf]

International Journal of *Molecular Sciences*

MDPI

Supplementary Materials

Table S1. Chemical components of Asian sand dust particles.

Component	Fraction (%)
SiO_2	52.13
Al_2O_3	15.80
Fe_2O_3	5.85
CaO	4.46
K_2O	2.57
MgO	2.43
Na_2O	1.59
TiO_2	0.83
P_2O_5	0.18
MnO	0.13
ZnO	0.05
BaO	0.02
SrO	0.02
Other elements	0.22
Loss ignition	13.72

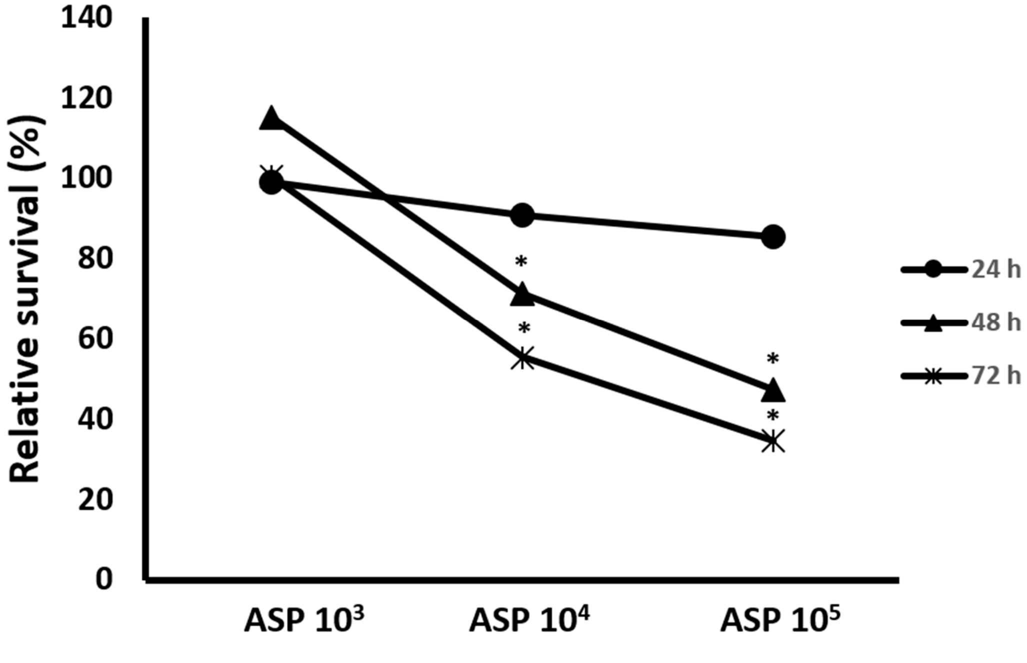

Figure S1. The effect of Aspergillus fumigatus on the survival of nasal epithelial cells. Primary nasal epithelial cells were incubated with 103/ml, 104/ml, and 105/ml of A. fumigatus conidia for 72 hours. Cell survival was significantly decreased above 104/ml of conidia at 48 and 72 h. *: $p < 0.05$ compared to without Aspergillus, n=5.

Methods S1. Determination of protease activity.

The protease activity of *A. fumigatus* was determined using a protease activity assay kit (Cayman, Ann Arbor, MI, USA). When nasal epithelial cells reached confluence, cells were incubated with *A. fumigatus* conidia (1 × 10^5/ml) for 2 h and then washed to remove the non-adherent fungi. Then, nasal epithelial cells were cultured in media, containing 50 μg/ml or 100 μg/ml of ASD for 24 h, 48 h, and 72 h. 100 μl of cell-free supernatants were placed in 96-well black plates with 100 μl of protease substrate for 20 min at RT. The value of protease activity was determined with an excitation wavelength of 485 nm and an emission wavelength of 520 nm using FLUOstar Optima (BMG Labtech. Ortenaukreis, Germany).

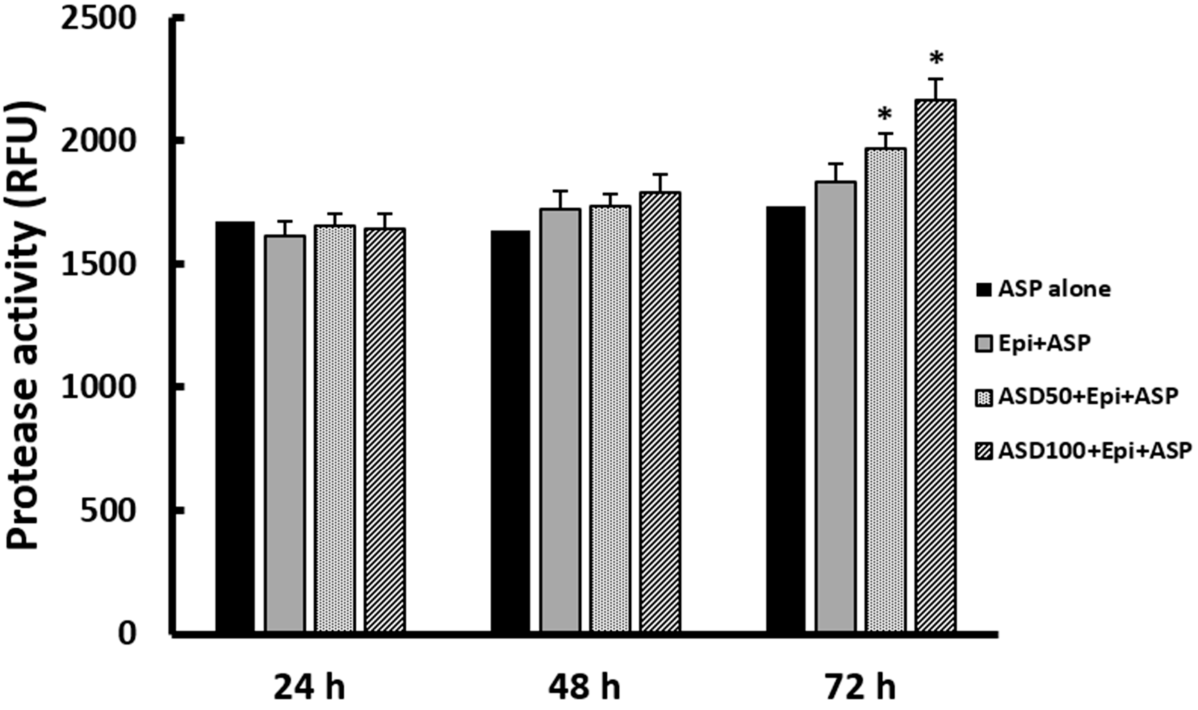

Figure S2. Protease activity of *Aspergillus fumigatus*. When the A. fumigatus cultured with 50 μg/ml or 100 μg/ml Asian sand dust (ASD) for 72 h, the protease activity was significantly increased at 72. *: $p < 0.05$ compared to ASP alone or ASP cultured without ASD, n=5.
